# Comparison of Topical Oxygen with Vacuum Assisted Closure in Wound Healing in a Low Resource Setting

**DOI:** 10.4314/ejhs.v32i5.11

**Published:** 2022-09

**Authors:** Tariq Akhtar Ansari, Sitanshu Barik, Pradeep Meena, Shobha Arora

**Affiliations:** 1 All India Institute of Medical Sciences, Rishikesh, India; 2 All India Institute of Medical Sciences, Deoghar, India

**Keywords:** Topical oxygen, vacuum assisted closure, Bates-Jensen wound assessment tool, wound healing

## Abstract

**Background:**

The aim of this pilot study is to obtain preliminary results comparing topical oxygen therapy (TOT) and vacuum assisted closure (VAC) in terms of its ability to accelerate wound healing.

**Methods:**

This non-randomised prospective study included patients with age 16–50 years, wound size ≥ 16cm^2^ and present below knee joint within seven days of occurrence. Bates-Jensen wound assessment tool (BWAT) was used for evaluation at 8-day interval along with percent area reduction at final follow up.

**Results:**

Mean number of cycles required in VAC and TOT group were 1.97 (range 1–3) and 2.1 (range 1–3) (each cycle of 5 days) per patient respectively. Percent area reduction was significantly higher in the VAC group (34±9.7%) than TOT (11.3±3.8%) group at final follow up (p<0.05). TOT patients had better improvement in epithelialization compared to VAC at last follow up. More extensive debridement was needed in patients of TOT than VAC. There was no significant difference between final score in both groups.

**Conclusions:**

TOT appears to be comparable to well-established VAC in treatment of fresh traumatic wounds below the knee joint. Further large scale, multicentric and randomised studies comparing both these modalities of treatment should be the way forward.

## Introduction

A wound site is characterised by disrupted vasculature as well as increased need for building blocks for its healing. The resultant hypoxia is due to increased demand and reduced supply of oxygen. In its healing, rate of availability of oxygen is considered to be one of most important factors (1). Wound healing has been shown to be accelerated by intermittent oxygen therapy which helps in synthesis of collagen as well as extracellular matrices(2). Studies have shown that adjunctive hyperbaric oxygen hastens wound healing and its secondary closure (3). The role of topical oxygen therapy (TOT) in healing of wounds, both acute and chronic, has also been well established with respect to hyperbaric oxygen (1). The easy availability as well its low cost are important advantages of topical oxygen.

Similarly, vacuum assisted closure (VAC) has become an important tool in wound healing since the 1990s (4). Microdeformation as well as macrodeformation play a role in this method. Microdeformation creates strain across healing tissues leading to increased cell proliferation with subsequent increase in growth factors. Fluid from the extracellular space is also removed leading to improved healing as well as the maintenance of a moist wound bed prevents desiccation of wound and increased granulation tissue formation (5). A commercially available VAC system is not economically viable in developing countries of Asia and Africa in terms of the cost of the equipment.

These two distinctive mechanisms of wound healing, namely hyperoxia (TOT) and strain (VAC) across the wound are widely used currently all over the globe. There is a lack of literature regarding the comparative efficacy of both the methods. The aim of this pilot study is to obtain preliminary results comparing TOT and VAC in terms of its ability to accelerate wound healing.

## Methods

It was a non-randomized prospective study performed at a tertiary level teaching hospital in a resource prone developing country from November 2017 to October 2019. Ethical clearance was obtained from the institutional review board (AIIMS/IEC/18/138). The research conforms to the guidelines set out in the Declaration of Helsinki.

Patients with age between 16 to 50 years, wound size equal or more than 16 cm^2^ (measured by maximum length multiplied by maximum width) and present below the knee joint which presented within seven days of occurrence were included in the study. Patients having any comorbidity, addiction, circumferential wound over the leg, associated vascular injury, preexisting venous ulcer disease and wound size less than 16 cm^2^ were excluded from study. Sixty consecutive patients who met the inclusion and exclusion criteria were divided into VAC and TOT group alternatively. After informed and written consent was obtained, wound was assessed and graded under Bates-Jensen wound assessment tool (BWAT) at 8 days interval and final score obtained when the wound bed was ready for secondary surgical procedure (6). The secondary surgical procedure planned was split thickness skin grafting. Healing rates were assessed by BWAT (Table 1). Percent area reduction was noted at final follow up which was calculated by the area of the wound at final follow up over the original area of the wound.

**Table 1:** Overview of the results obtained in the study

Variable	Topical oxygen therapy N=30)	Vacuum assisted closure (n=30)	p value
Mean age	28.8±9.4 years	30.5 ±12.3 years	>0.05
Gender	29 males, 1 female	26 males, 4 females	>0.05
Initial BWAT score	2.74±0.8	2.67±0.83	>0.05
Final BWAT score	2.16±0.85	2.12±0.98	>0.05
Number of cycles	2.1 (range 1–3)	1.97 (range 1–3)	>0.05
Percentage area reduction	11.3±3.8%	34±9.7%	**<0.05**

**Figure 1:**
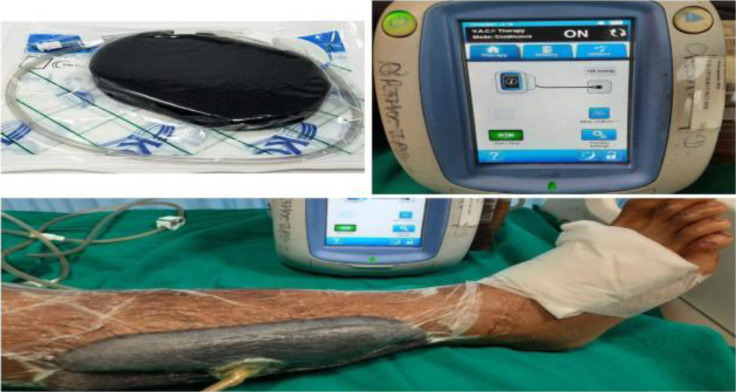
Components of VAC showing the sponge to be approximated over the wound, adhesive sealant, connecting tube and VAC machine along with its attachment in the patient.

**Figure 2:**
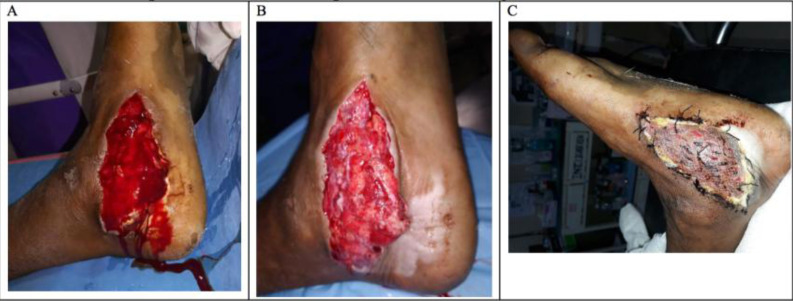
Depiction of a case under VAC with the A) initial injury, B) before secondary surgery and C) after final skin grafting.

**VAC application technique**: Wound was debrided and washed with normal saline under all aseptic precautions. A premanufactured foam of approximately equal wound size was applied over wound and sealed with the help of a sterilised adhesive sealant. A cruciate shape small opening made in the sealant and connected with vacuum creating device at 125 mm Hg pressure by tube (Figure 1). Discharge was collected in the transparent canister attached with VAC machine. This was kept applied continuously for a period of 5 days followed by a 3-day break. The cycle was discontinued when the wound was healthy and ready for secondary coverage (Figure 2). Repeat debridement was done in between the cycles, if needed.

**Figure 3:**
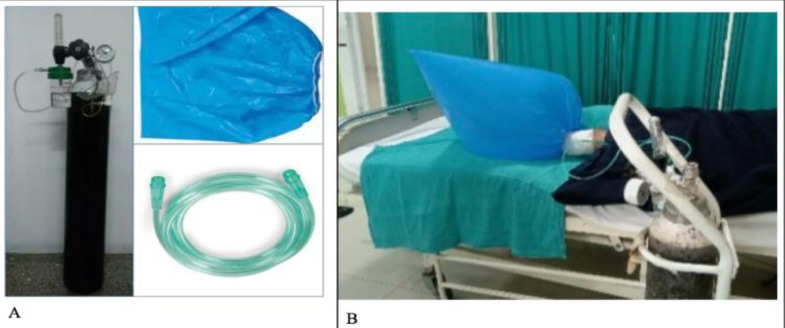
Components of TOT showing the oxygen cylinder, sterile cover and connecting tube along (A) with its attachment in the patient (B).

**Figure 4:**
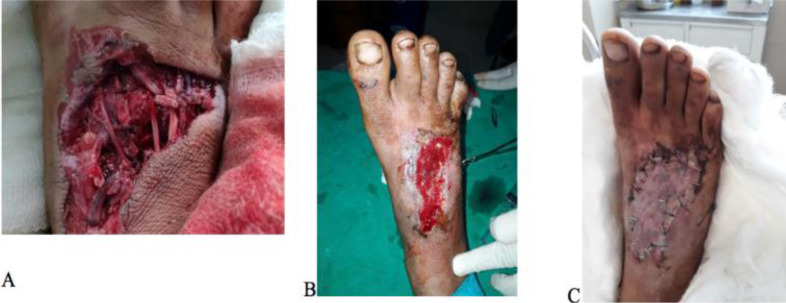
Depiction of a case under TOT with the A) initial injury, B) before secondary surgery and C) after final skin grafting.

**TOT application technique**: Wound was debrided and washed with normal saline under all aseptic precautions. A sterile C-arm covering sheet was used for wound coverage and sealed with adhesive tape circumferentially over the leg. It was connected to an oxygen cylinder by a connecting tube (Figure 3). The sealed cover was filled with oxygen at the rate of 5–6 litres per minute. Each cycle consisted of TOT for 5 days consecutively of 60 minutes every day followed by a 3-day break. The process was discontinued when the floor of the wound was ready for secondary coverage (Figure 4). Repeat debridement was done in between the cycles, if needed.

Descriptive analysis of the data was done in terms of mean and standard deviation. Wilcoxon test was used for non-parametric data analysis. The analysis of difference in significance was done by Friedman Q test. ANOVA test was used for comparison between time duration and percent area reduction in individual cycles. Post hoc Bonferroni correction was applied for the multiple comparisons in BWAT. Statistical calculations were performed by SPSS version 22.

## Results

Sixty patients were included in the current study with 30 each in VAC (26 males, 4 females, mean 30.5 ±12.3 years) and TOT (29 males, 1 female, mean 28.8±9.4 years) group (Table 1).

All the wounds were result of road traffic accidents. The mean number of cycles required in VAC and TOT group were 1.97 (range 1–3) and 2.1 (range 1–3) cycles per patient respectively before being ready for the secondary surgical procedure. There was significant difference in all the factors of BWAT in ptients treated with VAC except for a) colour of the skin in surrounding tissue b) peripheral tissue oedema and c) peripheral tissue induration. In the TOT group, a) the size of the wound and b) peripheral tissue induration did not improve significantly, although all the other parameters improved (Table 2). Percent area reduction was significantly higher in the VAC group (34±9.7%) than TOT (11.3±3.8%) group at final follow up (p<0.05). TOT patients had better improvement in epithelialization compared to VAC at last follow up. More extensive debridement was needed in patients of TOT than VAC. It was also noted that skin colour of surrounding areas was closer to the ethnic skin colour in VAC. There was no significant difference between final score in both groups (Table 2). No adverse event was noted in either group during the course of treatment nor was there any switch from one group to other.

**Table 2:** Results of the study showing the comparison between initial and final scores of both TOT and VAC group and in comparison, to each other (* Post hoc Bonferroni correction for multiple comparisons)

Variables		Bates-Jensen wound assessment tool Scores												
		
				VAC						TOT				
		Initial score	Final score	p value	p value*	SD	95% CI	Initial score	Final score	p value	p value*	SD	95% CI	p value
1	Size	3.86	3.36	**< 0.001**	**0.023**	0.95	0.35	3.63	3.36	0.07	0.057	0.89	0.33	
2	Depth	3.13	2.46	**< 0.001**	**0.037**	0.73	0.27	3.06	2.46	**< 0.001**	**0.034**	0.67	0.25	
3	Edges	2.86	1.93	**< 0.001**	**0.028**	0.64	0.24	2.70	1.96	**< 0.001**	**0.021**	0.87	0.32	
4	Undermining	2.50	1.50	**< 0.001**	**0.015**	0.95	0.35	2.50	1.70	**< 0.001**	**0.041**	0.93	0.34	
5	Necrotic tissue type	2.06	1.60	**< 0.001**	**0.028**	0.55	0.20	2.10	1.70	**< 0.001**	**0.003**	0.98	0.36	
6	Necrotic tissue amount	2.06	1.60	**< 0.001**	**0.018**	0.82	0.30	2.23	1.63	**< 0.001**	**0.029**	0.88	0.32	
7	Exudate type	2.50	1.66	**< 0.001**	**0.002**	0.69	0.25	2.36	1.70	**< 0.001**	**0.004**	0.81	0.30	
8	Exudate amount	2.66	1.83	**< 0.001**	**0.005**	0.83	0.31	2.70	1.90	**< 0.001**	**0.043**	0.92	0.34	
9	Skin colour surrounding wound	2.83	1.66	0.06	0.06	0.91	0.34	2.66	2.13	**< 0.001**	**0.021**	0.84	0.31	
10	Peripheral tissue edema	3.06	1.83	0.06	0.06	1.09	0.40	2.33	1.80	**< 0.001**	**0.001**	0.70	0.26	
11	Peripheral tissue induration	1.53	1.10	**< 0.001**	0.07	0.65	0.24	1.56	1.36	**< 0.001**	0.09	0.53	0.20	
12	Granulation tissue	2.76	2.06	**< 0.001**	**0.012**	0.84	0.31	2.80	1.86	**< 0.001**	**0.023**	0.95	0.35	
13	Epithelialization	5	5	-	-	0	-	5.0	4.63	**< 0.001**	**0.031**	0.56	0.21	
	Mean score	2.67	2.12					2.74	2.16					0.23
Standardized Mean difference	0.74				0.66						0.12			

## Discussion

Healing of wound essentially includes three overlapping phases, namely, inflammation, proliferation and remodelling. After initial trauma, haemostatic mechanisms to reduce blood loss are coagulation and vasoconstriction (7). Oxygen delivery at this stage is compromised and is dependent on diffusion from surrounding tissues and atmosphere. Initial transient hypoxia in vitro has been shown to improve angiogenesis, cell proliferation and collagen formation via different growth factors (8). But as healing process of inflammation starts, there in increased oxygen demand by the cells which aren't met eventually because of disruption of vascular supply as mentioned above, leading to anaerobic metabolism and lactic acidosis (7). The accumulated lactic acidosis is also not cleared off due to the deficient vascularity. This acidic environment also hampers healing. Hence, when this transient hypoxia is prolonged, wound healing is impeded. It explains the need for supplemental oxygen in acute phases of wound healing. After this has enabled the neovascularisation, adjunct therapies like hyperbaric oxygen or VAC help in speeding up the healing process. The supplemental oxygen in the initial phases has also seen to reduce the secondary infections (9).

VAC helps in wound healing, by different but overlapping mechanisms as TOT. It improves the macrocirculation as well as microcirculation. The macrocirculation is improved by the strain that it applies across the tissues and subsequent effect on circulation by negative pressure. There is also reduction of interstitial oedema leading to local decompression without systemic haemostatic effects which increases the microcirculation (10). Due to the improved circulation, the delivery of oxygen to the healing tissues is increased leading to the effects as described above namely, angiogenesis, cell proliferation and collagen formation. Improved circulation also leads to better localisation of the humoral as well as cellular immunity which keeps any infection in check (10). VAC also leads to reduction in wound size owing to the strain it applies across the edges which reduces the burden of the secondary procedure for coverage (11).

These two distinct but overlapping mechanisms have not proven to be superior to each other in this study. Both the study groups did not have any significant difference at the end point before the patient proceeded to the secondary surgery for coverage. Although, there are a few subtle differences which need mention. The wound size showed significant reduction in the VAC group, unlike in TOT group. Whether this size reduction reduces the burden of the secondary surgery, should be a matter for future studies. This study also showed there is minimal effect of VAC on the skin colour of peripheral tissue, its oedema and induration. Whereas in TOT, the skin colour of peripheral tissues approached that of the native colour. Peripheral tissue induration did not improve in both which may suggest that both these techniques have minimal anti-inflammatory activities in the surrounding tissue. This may be attributed to the fact that TOT is applied for a wide area inside the sealed cover whereas VAC has its effect over the wound surface only. It was also noted epithelialization was inferior in VAC which can be due to the continuous strain being applied by it which does not let the newly formed vessels as well as epithelium to hold on for long.

Cost-effectiveness is another major factor which has not been considered in this study. In the developing countries of Asia and Africa, this can be a game changer over which treatment method is preferred considering the fact that this study doesn't prove any of the method to be superior. Studies have focussed on the high costs involved in VAC and its inability to be used on a regular basis in countries where the poor socioeconomic status of the patient makes it difficult (12). Though numerous studies have been conducted which use low cost VAC devices, large scale multicentric prospective studies are lacking regarding its efficacy (13). TOT in comparison to VAC, is cost effective considering the components which are required for its use(14,15). A sterile sealant, like a C arm cover, as well as continuous oxygen supply are ubiquitous in any hospital catering to orthopaedic and plastic surgery patients. Cost benefit studies are planned to be conducted in a full-fledged manner on the basis of the results of this pilot study.

This study would help the researchers in planning further large-scale randomised studies. The strength of this study is its prospective comparative nature and its use of an objective criteria like BWAT. The scoring system has been proved to be having good inter-observer as well as intra-observer correlation (6). Though initially designed for pressure sores, this score has been expanded to assess acute wounds including posttraumatic and post-surgical wounds (6). This study is one of the first studies comparing VAC and TOT, with two distinct but overlapping mechanisms of action, prospectively. The small sample size as well as non-randomisation are the limitations of this study. The effect of VAC or TOT on the final outcome in terms of secondary surgery for coverage has not been studied here. The end point of the study, which was when the bed is healthy for a secondary procedure, should be expanded to the final outcome till the patient obtains a coverage which doesn't require further interventions. This study misses out on conducting a cost analysis study. Adequate precautions in terms of healthcare worker and patient education are needed in TOT to prevent any fire hazard due to the use of oxygen. Also, the amount of oxygen in the sealant cover was not measured, which is a limitation in this study preventing any widescale guidelinesto be formulated. Further large scale, multicentric and randomised studies comparing both these modalities of treatment should be the way forward.

To conclude, TOT appears to be comparable to well-established VAC in treatment of fresh traumatic wounds below the knee joint. The technical use, cost effectiveness and the absence of any systemic side effects can make topical oxygen therapy to be an ideal future modality in fresh traumatic wounds in not only the developing countries but globally. At the end, it should also be noted that even though TOT has the potential for improving treatment where funding is limited, this study also points to the use of relatively hightech treatments which may be available in a tertiary referral centre but which would not be available elsewhere in other parts of the country.
